# Supplementary material for: Regional political climate’s moderating role in the association between political conservatism and COVID-19 vaccine hesitancy in the United States
Source: PLoS One. 2026 Feb 3;21(2):e0342063. doi: 10.1371/journal.pone.0342063 (PMC12867218; doi:10.1371/journal.pone.0342063)
Supplement: S1 File — (DOCX) [file pone.0342063.s001.docx]

# **S1 File:** **Items from Life Experiences and COVID-19 – Qualtrics**

*(Attention Check 1)*

**We care about the quality of this survey data. For us to get the most accurate measures of your opinions, it is important that you provide thoughtful answers to each question in**

**this survey. Do you commit to providing thoughtful answers to the questions in this survey?**

I can't promise either way

Yes, I will

No, I will not

**What is your gender (check all that apply)?**

Female

Male

Nonbinary

Transgender

I prefer to specify __________________________________________________

**What is your age?**__________________________________________________

**What is your racial/ethnic background (check all that apply)?**

American Indian or Alaska Native

Asian

Black or African American

Hispanic or Latinx

Native Hawaiian or Other Pacific Islander

White

I prefer to specify __________________________________________________

**What state is your primary residence (where you live most of the time)?**

Alabama

Alaska

Arizona

California

Colorado

Connecticut

Delaware

District of Columbia

Florida

Georgia

Hawaii

Idaho

Illinois

Indiana

Iowa

Kansas

Kentucky

Louisiana

Maine

Maryland

Massachusetts

Michigan

Minnesota

Mississippi

Missouri

Montana

Nebraska

Nevada

New Hampshire

New Jersey

New Mexico

New York

North California

North Dakota

Ohio

Oklahoma

Oregon

Pennsylvania

Rhode Island

South California

South Dakoha

Tennessee

Texas

Utah

Vermont

Virginia

Washington

West Virginia

Wisconsin

Wyoming

Other, please specify__________________________________________________

**What is the zip code of your primary residence?**_______________________________

**What is the highest level of education you have completed?**

Below primary school

Primary school

Secondary school

High school graduate

Trade/technical/vocational training

Some college

Bachelor's degree

Master's degree

Professional degree

Doctoral degree

**What is your annual household income?**

under $10,000

$10,000 to $19,999

$20,000 to $29,999

$30,000 to $39,999

$40,000 to $49,999

$50,000 to $59,999

$60,000 to $69,999

$70,000 to $79,999

$80,000 to $89,999

$90,000 to $99,999

$100,000 to $109,999

$110,000 to $119,999

$120,000 to $129,999

$130,000 to $139,999

$140,000 to $149,999

$150,000 to $159,999

$160,000 to $169,999

$170,000 to $179,999

$180,000 to $189,999

$190,000 to $199,999

$200,000 or over

**Slide the bar to indicate your political orientation on social issues (for example, abortion, gun rights, gay rights).**

On **social issues** I am...

|  | Strongly Liberal | Strongly Conservative |
| --- | --- | --- |

|  | 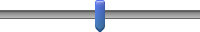 |
| --- | --- |

**Slide the bar to indicate your political orientation on economic issues (for example, taxation, government spending).**

 On **economic issues** I am...

|  | Strongly Liberal | Strongly Conservative |
| --- | --- | --- |

| () | 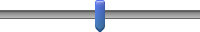 |
| --- | --- |

*(Attention Check 2)*

**Please select "purple" below to show you are paying attention.**

green

red

purple

black

**Have you received a COVID-19 vaccine?**

Yes

No

I don't know

Please rate the extent to which you agree or disagree with each of the following statements about **COVID-19**

Disagree Strongly

Disagree Somewhat

Neutral

Agree Somewhat

Agree Strongly

I believe in the value of COVID-19 vaccination

I believe that COVID-19 vaccines are necessary for adults

I believe that the benefits of COVID-19 vaccination outweigh the potential risks

I believe that COVID-19 vaccines are very effective in protecting me from getting COVID-19

I haven’t had a vaccine as an adult so far, so I don’t need the COVID-19 vaccine

I fear the immediate complications of the COVID-19 vaccine (such as allergic reactions)

I fear the potential impact of the COVID-19 vaccine on my health in the future

**If I needed to get a COVID-19 vaccine or booster, I could easily get it**

Disagree Strongly

Disagree Somewhat

Neutral

Agree Somewhat

Agree Strongly

**Have you received a COVID-19 vaccine** **booster**?

Yes

No

I don't know

**How much do you agree or disagree with each of the following statements about the COVID-19 vaccine booster?**

Disagree Strongly

Disagree Somewhat

Neutral

Agree Somewhat

Agree Strongly

I worry about serious adverse reaction after receiving the COVID-19 vaccine booster dose

I believe it is safe to the receive COVID-19 vaccine booster

Boosters are effective against COVID-19 variants

I have high level of fear associated with receiving the COVID-19 vaccine booster

It is important to get COVID-19 boosters when they are recommended

*(Attention Check 3)*

**The following question is to verify that you are a real person. Please type the word TOPS into the box below.** ______________________________________________________________
