# Supplementary material for: Regional political climate’s moderating role in the association between political conservatism and COVID-19 vaccine hesitancy in the United States
Source: PLoS One. 2026 Feb 3;21(2):e0342063. doi: 10.1371/journal.pone.0342063 (PMC12867218; doi:10.1371/journal.pone.0342063)
Supplement: S2 File — (DOCX) [file pone.0342063.s002.docx]

# **S2 File: Confirmatory Factor Analysis**

**Vaccine Hesitancy**

I believe in the value of COVID-19 vaccination

I believe that COVID-19 vaccines are necessary for adults

I believe that the benefits of COVID-19 vaccination outweigh the potential risks

I believe that COVID-19 vaccines are very effective in protecting me from getting COVID-19

I haven’t had a vaccine as an adult so far, so I don’t need the COVID-19 vaccine

I fear the immediate complications of the COVID-19 vaccine (such as allergic reactions)

I fear the potential impact of the COVID-19 vaccine on my health in the future

First, we ran confirmatory factor analysis (CFA) on the vaccine hesitancy items from the Attitudes towards Adult Vaccination Scale [1] loading all seven items onto one latent factor. This model fit poorly (CFI = .854, TLI = .781, RMSEA = .208 95%CI[.193, .224]. The first four items exhibited high factor loading (λ_1_ = .916, λ_2_ = .865, λ_3_ = .849, λ_4_ = .842), while the other three items were below the accepted .50 threshold [2] (λ_5_ = .488, λ_6_ = .355, λ_7_ = .328). We the ran CFA with the first 4 items loading onto one latent factor, which fit the data adequately (CFI = .997, TLI = .990, RMSEA = .071 95%CI[.031, .117]; λ_1_ = .916, λ_2_ = .865, λ_3_ = .849, λ_4_ = .842). Next, we tested for structural invariance across both social and economic conservatism. To do this we created a median spilt for economic conservatism (median score = 5, N = 403 participants at or below median income, N = 387 participants above median income) and social conservatism (median score = 5, N = 438 participants at or below median income, N = 353 participants above median income). We tested the CFA model for structural invariance by comparing it across levels of economic conservatism and social conservatism. Neither of these comparisons were significant ((Χ^2^Δ = 4.340, p = .227; AIC_configural_ = 8613.5, AIC_constrained_ = 8611.8 for economic conservatism; Χ^2^Δ = 5.291, p = .152; AIC_configural_ = 8608.6, AIC_constrained_ = 8607.8 for social conservatism). Given this, the scale score for vaccine hesitancy was created by averaging the first 4 items.

CFA for the booster hesitancy items was performed by loading all five items onto one latent factor. Model fit poorly (CFI = .822, TLI = .643, RMSEA = .349 95%CI[.324, .376]. Items 2, 3, and 5 exhibited high factor loading (λ_2_ = .890, λ_3_ = .916, λ_5_ = .924), while the other two items were below the accepted .50 threshold [2] (λ_1_ = .403, λ_4_ = .325). We the ran CFA with items 2, 3, and 5 loading onto one latent factor, which fit the data adequately (CFI = 1.00, TLI =1.00, RMSEA = .000 95%CI[.000, .000]; λ_2_ = .884, λ_3_ = .917, λ_5_ = .928). Next, we tested for structural invariance across both social and economic conservatism. Neither of these comparisons were significant ((Χ^2^Δ = 2.534, p = .282; AIC_configural_ = 6277.4, AIC_constrained_ = 6275.9 for economic conservatism; Χ^2^Δ = 1.353, p = .508; AIC_configural_ = 6272.4, AIC_constrained_ = 6269.8 for social conservatism). Given this, the scale score for vaccine hesitancy was created by averaging the first three items.

# **References**

1. Tsimtsiou Z, Tsiligianni I, Papaioannou A, Gougourelas D, Kolokas K, Gkizlis V, et al. Understanding what people think and feel about adult vaccinations and the associated barriers in Greece: Development and validation of the attitude towards adult vaccination (ATAVAC) scale. Health Soc Care Community. 2021;29(3):818–28.

2. Hair, J. F., Black, W. C., Babin, B. J., & Anderson, R. E. (2019). Multivariate Data Analysis (8th ed.). Cengage.
