# Supplementary material for: Regional political climate’s moderating role in the association between political conservatism and COVID-19 vaccine hesitancy in the United States
Source: PLoS One. 2026 Feb 3;21(2):e0342063. doi: 10.1371/journal.pone.0342063 (PMC12867218; doi:10.1371/journal.pone.0342063)
Supplement: S3 Table — (DOCX) [file pone.0342063.s003.docx]

# S3 Table: Participant Distribution by State

|  | **Number of Participants** |
| --- | --- |
| Alabama | 0 |
| Alaska | 0 |
| Arizona | 11 |
| Arkansas | 0 |
| California | 50 |
| Colorado | 15 |
| Connecticut | 0 |
| Delaware | 1 |
| District of Columbia | 2 |
| Florida | 66 |
| Georgia | 38 |
| Hawaii | 1 |
| Idaho | 6 |
| Illinois | 43 |
| Indiana | 11 |
| Iowa | 3 |
| Kansas | 3 |
| Kentucky | 1 |
| Louisiana | 0 |
| Maine | 0 |
| Maryland | 20 |
| Massachusetts | 5 |
| Michigan | 16 |
| Minnesota | 13 |
| Mississippi | 9 |
| Missouri | 4 |
| Montana | 1 |
| Nebraska | 4 |
| Nevada | 13 |
| New Hampshire | 0 |
| New Jersey | 25 |
| New Mexico | 5 |
| New York | 68 |
| North Caroline | 27 |
| North Dakoha | 1 |
| Ohio | 38 |
| Oklahoma | 5 |
| Oregon | 5 |
| Pennsylvania | 26 |
| Rhode Island | 0 |
| South Carolina | 14 |
| South Dakota | 2 |
| Tennessee | 21 |
| Texas | 76 |
| Utah | 9 |
| Vermont | 0 |
| Virginia | 0 |
| Washington | 6 |
| West Virginia | 0 |
| Wisconsin | 0 |
| Wyoming | 0 |
