# Supplementary material for: Regional political climate’s moderating role in the association between political conservatism and COVID-19 vaccine hesitancy in the United States
Source: PLoS One. 2026 Feb 3;21(2):e0342063. doi: 10.1371/journal.pone.0342063 (PMC12867218; doi:10.1371/journal.pone.0342063)
Supplement: S4 File — (DOCX) [file pone.0342063.s004.docx]

**S4 File. Results for All Regression Models with Control Variables.**

**Table A. Linear regression for vaccine hesitancy with main effects political conservatism and conservative regional climate, the interaction between political conservatism and conservative regional climate, United States, 2023.**

| **Variable** | **Model 1: Control Variables^1^**  **(n = 669)** | | |  | **Model 2: Main Effects^2^**  **(n = 664)** | | |  | **Model 3: Interaction^3^**  **(n = 664)** | | |
| --- | --- | --- | --- | --- | --- | --- | --- | --- | --- | --- | --- |
|  | **B** | **95% CI** | ***P*** |  | **B** | **95% CI** | ***p*** |  | **B** | **95% CI** | ***p*** |
| Age | -.06 | -.13, .01 | .116 |  | -.09 | -.17, -.02 | .010 |  | -.10 | -.32, -.16 | .006 |
| Female (vs. male) | .00 | -.13, .14 | .954 |  | .00 | -.13, .14 | .941 |  | .01 | -.19, .07 | .897 |
| Education | -.12 | -.19, -.04 | .002 |  | -.09 | -.17, -.02 | .008 |  | -.10 | -.14, -.01 | .006 |
| Income | -.05 | -.13, -.02 | .180 |  | -.05 | -.12, -.03 | .215 |  | -.05 | -.12, -.02 | .209 |
| Black (vs. White) | -.25 | -.40, -.11 | < .001 |  | -.17 | -.31, -.03 | .020 |  | -.16 | -.31, -.05 | .023 |
| Vaccine Access | -.49 | -.56, -.42 | <.001 |  | -.44 | -.50, -.37 | <.001 |  | -.43 | -.42, -.29 | <.001 |
| Political Conservatism |  |  |  |  | .22 | .16, .28 | < .001 |  | .23 | .23, .35 | < .001 |
| Conservative Political Climate |  |  |  |  | .11 | .05, .18 | .001 |  | .11 | .03, .17 | .002 |
| Conservatism * Conservative Climate |  |  |  |  |  |  |  |  | .09 | .00, .13 | .003 |

^1^Control model adjusted *R^2^* = .294, *F*(667) = 47.45, *p* <.001

^2^ Main effects model adjusted *R^2^* = .347, *F*(655) = 45.11, *p* <.001

^3^ Interaction model adjusted *R^2^* = .355, *F*(654) = 41.54, *p* <.001

**Table B. Linear regression for booster hesitancy with main effects political conservatism and conservative regional climate, the interaction between political conservatism and conservative regional climate, United States, 2023.**

| **Variable** | **Model 1: Controls**  **(n = 669)** | | |  | **Model 2: Main Effects**  **(n = 664)** | | |  | **Model 3: Interactions**  **(n = 664)** | | |
| --- | --- | --- | --- | --- | --- | --- | --- | --- | --- | --- | --- |
|  | **B** | **95% CI** | ***p*** |  | **B** | **95% CI** | ***p*** |  | **B** | **95% CI** | ***p*** |
| Age | -.07 | -.14, .01 | .093 |  | -.10 | -.17, -.02 | .012 |  | -.10 | -.18, -.03 | .007 |
| Female (vs. male) | .03 | -.12, .17 | .719 |  | .02 | -.12, .16 | .747 |  | .03 | -.11, .17 | .704 |
| Education | -.10 | -.17, -.02 | .014 |  | -.08 | -.15, -.00 | .046 |  | -.08 | -.15, -.01 | .036 |
| Income | -.08 | -.16, -.00 | .046 |  | -.07 | -.15, .01 | .072 |  | -.07 | -.15, .01 | .069 |
| Black (vs. White) | -.24 | -.39, -.09 | .002 |  | -.16 | -.31, -.01 | .035 |  | -.16 | -.30, -.01 | .040 |
| Vaccine Access | -.41 | -.49, -.34 | <.001 |  | -.37 | -.44, -29 | <.001 |  | -.36 | -.44, -.29 | < .001 |
| Political Conservatism |  |  |  |  | .18 | .11, .24 | < .001 |  | .18 | .12, .25 | < .001 |
| Conservative Political Climate |  |  |  |  | .11 | .04, .19 | .002 |  | .11 | .04, .18 | .003 |
| Conservatism * Conservative Climate |  |  |  |  |  |  |  |  | .11 | .04, .17 | .003 |

^1^ Control model adjusted *R^2^* = .225, *F*(667) = 33.20, *p* <.001

^2^ Main effects model adjusted *R^2^* = .262, *F*(655) = 30.43, *p* <.001

^3^ Interactions model adjusted *R^2^* = .271, *F*(654) = 28.39, *p* <.001

**Table C. Logistic regression for vaccine status with main effects political conservatism and conservative regional climate, the interaction between political conservatism and conservative regional climate, United States, 2023.**

| **Variable** | **Model 1: Controls**  **(n = 668)** | | |  | **Model 2: Main Effects**  **(n = 663)** | | |  | **Model 3: Interactions**  **(n = 663)** | | |
| --- | --- | --- | --- | --- | --- | --- | --- | --- | --- | --- | --- |
|  | **OR** | **95% CI** | ***p*** |  | **OR** | **95% CI** | ***p*** |  | **OR** | **95% CI** | ***p*** |
| Age | 1.30 | 1.04, 1.64 | .030 |  | 1.44 | 1.14, 1.84 | .002 |  | 1.44 | 1.14, 1.84 | .002 |
| Female (vs. male) | 1.45 | .96, 2.18 | .103 |  | 1.55 | 1.02, 2.38 | .060 |  | 1.55 | 1.02, 2.38 | .060 |
| Education | 1.56 | 1.26, 1.96 | < .001 |  | 1.52 | 1.22, 1.92 | < .001 |  | 1.52 | 1.22, 1.92 | < .001 |
| Income | 1.28 | 1.01, 1.63 | .080 |  | 1.29 | 1.01, 1.67 | .086 |  | 1.29 | 1.01, 1.67 | .087 |
| Black (vs. White) | 1.39 | .90, 2.15 | .163 |  | 1.13 | .71, 1.80 | .631 |  | 1.13 | .71, 1.80 | .632 |
| Vaccine Access | 1.68 | 1.39, 2.04 | <.001 |  | 1.55 | 1.28, 1.90 | <.001 |  | 1.55 | 1.28, 1.90 | <.001 |
| Political Conservatism |  |  |  |  | .68 | .54, .84 | < .001 |  | .68 | .54, .84 | < .001 |
| Conservative Political Climate |  |  |  |  | .73 | .58, .90 | .004 |  | .72 | .58, .90 | .004 |
| Conservatism * Conservative Climate |  |  |  |  |  |  |  |  | 1.02 | .82, 1.27 | .833 |

^1^Control model AIC = 655.35

^2^Main effects model AIC = 627.73

^3^Interactions model AIC = 629.70

**Table D. Logistic regression for booster status with main effects political conservatism and conservative regional climate, the interaction between political conservatism and conservative regional climate, United States, 2023.**

| **Variable** | **Model 1: Controls**  **(n = 660)** | | |  | **Model 2: Main Effects**  **(n = 655)** | | |  | **Model 3: Interactions**  **(n = 655)** | | |
| --- | --- | --- | --- | --- | --- | --- | --- | --- | --- | --- | --- |
|  | **OR** | **95% CI** | ***p*** |  | **OR** | **95% CI** | ***p*** |  | **OR** | **95% CI** | ***p*** |
| Age | 1.67 | 1.37, 2.05 | < .001 |  | 1.79 | 1.46, 2.20 | < .001 |  | 1.81 | 1.48, 2.24 | < .001 |
| Female (vs. male) | 1.16 | .81, 1.66 | .427 |  | 1.19 | .82, 1.71 | .374 |  | 1.17 | .81, 1.70 | .403 |
| Education | 1.36 | 1.12, 1.66 | .005 |  | 1.32 | 1.09, 1.61 | .013 |  | 1.33 | 1.09, 1.62 | .012 |
| Income | 1.47 | 1.21, 1.81 | < .001 |  | 1.42 | 1.16, 1.75 | .002 |  | 1.42 | 1.16, 1.75 | .002 |
| Black (vs. White) | 1.30 | .90, 1.91 | .158 |  | 1.11 | .75, 1.65 | .573 |  | 1.11 | .75, 1.65 | .589 |
| Vaccine Access | 1.61 | 1.24, 1.96 | < .001 |  | 1.50 | 1.23, 1.82 | < .001 |  | 1.50 | 1.24, 1.83 | < .001 |
| Political Conservatism |  |  |  |  | .83 | .69, .99 | .034 |  | .82 | .69, .98 | .022 |
| Conservative Political Climate |  |  |  |  | .76 | .63, .92 | .002 |  | .77 | .63, .93 | .004 |
| Conservatism * Conservative Climate |  |  |  |  |  |  |  |  | .83 | .69, .99 | .026 |

^1^Control model AIC = 794.34

^2^Main effects model AIC = 781.67

^3^Interactions model AIC = 779.58
