# Supplementary material for: Regional political climate’s moderating role in the association between political conservatism and COVID-19 vaccine hesitancy in the United States
Source: PLoS One. 2026 Feb 3;21(2):e0342063. doi: 10.1371/journal.pone.0342063 (PMC12867218; doi:10.1371/journal.pone.0342063)
Supplement: Attachment — Submitted filename: Response to Reviewers.docx [file pone.0342063.s007.docx]

**Additional Editor Comments:**

The authors have fully addressed my comments about the methodology. However, upon checking the results and discussion, there are some issues that need to be addressed before the manuscript can be accepted for publication. Notably, the total of percentages should be 100 for each variable on Table 1.

**Author response: We rounded to the nearest 10^th^ so that all percents would add to 100%.**

Did you exclude the Simpson paradox given the lack of effect modification of main study results by age, sex, ethnicity and so on?

**Author response: We controlled for demographic covariates; however, we did not formally test for effect modification across these variables. As such, we cannot fully exclude the possibility of Simpson’s paradox. We have clarified this point in the limitations by adding the following statement: “While our analyses controlled for demographic covariates commonly associated with vaccine hesitancy, we did not formally assess effect modification by these factors. Consequently, the reported pooled associations should be interpreted as average effects across the sample, and we cannot exclude the possibility that heterogeneity across demographic subgroups could produce aggregation effects such as Simpson’s paradox.”**

I suggest separating results of moderation analysis from results of main analyses in tables 3-6.

**Author response: We restructured these table to make them easier to read. We moved the original tables to the S4 file and now do not include the control variables in the in-text tables. We also separated the moderation analysis from main effects analysis.**

Could you simplify the titles of those tables without mentioning the covariates in titles?

**Author response: Thank you for this suggestion, the covariates were removed from the table title.**

Regarding the titles of tables 5 and 6, did you wish to say linear rather than logistic regression?
**Author response: Tables 5 and 6 are both results from logistic regression, while table Tables 3 and 4 are results from linear regression.**

**Review Comments to the Author:**

Reviewer #1: Dear Author(s),
This revised manuscript presents a high-quality, methodologically sound, and policy-relevant study that makes a clear contribution to the existing literature. The improvements made in response to prior feedback—especially the enhanced theoretical framing, clearer explanation of variables, and expanded discussion—demonstrate great effort and scholarly care. The paper is now well-structured, coherent, and supported by strong empirical evidence. This is a strong, well-developed manuscript. I commend the authors for their thorough revisions and recommend the paper for publication in its current form.
**Author response: Thank you for your feedback and taking the time to review our revised manuscript.**

Reviewer #3: The authors reflect each of all my review comments logically and rigorously. Therefore, I believe this manuscript is publishable to international readers. I hope this manuscript is cited and read by many readers across the globe.

**Author response: Thank you for your feedback and taking the time to review our revised manuscript.**

Reviewer #4: Dear Editor,
Thank you for having given to me the opportunity to review the manuscript titled "The Moderating Role of Regional Political Climate on the Association Between Political Conservatism and COVID-19 Vaccine Hesitancy in the United States." This article investigates whether local political climate, measured by Republican vote share at the ZIP code level in the 2020 presidential election, moderates the association between self-reported political conservatism and COVID-19 vaccine hesitancy and uptake. The authors find that progressives exhibit low hesitancy and relatively high vaccine uptake across all contexts, while conservatives are less hesitant and more likely to receive a response in more progressive environments than in conservative ones.

The topic is highly timely and important, and the manuscript has several strengths: the research question is clearly articulated, the analytical strategy is transparent, and the psychometric properties of the hesitancy scales are robust. The provision of open data and materials, often overlooked in survey-based research, is certainly an excellent foundation.
Although the article has improved, thanks in part to previous comments, I believe its overall contribution is modest, and several conceptual and methodological issues substantially limit the conclusions that can be drawn from the results.

First, the central model, according to which conservatism and a conservative regional context are associated with greater COVID-19 vaccine hesitancy, is already well documented in the literature (I don't think it's necessary to cite previous studies published on the topic here, given their vast and therefore easily accessible nature). The manuscript's claim to novelty likely rests on two aspects. (a) The interaction between ideology and local political climate. These interaction effects are statistically significant but small, derived from self-reported cross-sectional data in a non-probability sample and estimated with a crude ecological proxy for the context. The article is not fully convinced that this adds more than an incremental refinement to what is already known. (b) Certainly more interesting, and the main contribution to the article, is to have identified how those politically aligned to the right, or at least with conservative views, tend to be less hesitant, and therefore more willing, to vaccinate if they live in more progressive areas. This is likely related to social pressure from the context, which would explain this result, which is certainly relevant to note.

**Author response: Thank you for this feedback, we have emphasized this critical point more clearly in the discussion.**

Second, the operationalization of "political climate" as a Republican vote share is understandable but rather limited. This variable almost certainly combines political norms with structural and demographic characteristics such as urbanity, racial composition, socioeconomic status, and access to healthcare. No ecological controls are included in the control variables, and the analysis does not account for clustering by geographic area. This raises serious concerns about ecological confounding and underestimated standard errors, weakening any interpretation of climate as an independent contextual influence on individuals.

**Author response: This is certainly a limitation to this common strategy for conceptualizing political climate, and one that we have addressed in the study limitations.**

Third, the sampling strategy and the resulting analytic sample limit generalizability. The study uses a non-probabilistic online panel, excludes a large fraction of initial respondents, and then further restricts the sample to Black and White participants with valid zip codes. These choices are not unreasonable, but they require a much more explicit discussion of the selection processes and the limitations of demographic inference than currently provided.
**Author response: We have explained the process in the methods section and discussed the limitations in the discussion. Additionally, we have included the citation for the initial study that guided the data collection methodology. We feel this is transparent and sufficient for readers to understand the data collection process and resulting limitations.**

Finally, some aspects of the structure and interpretation require revision. In several places, the manuscript lapses into causal and perhaps politically biased language that is not supported by cross-sectional observational data, as it should be. The suggested implications for the targeting of conservatives in progressive regions are potential and should be clearly formulated as hypotheses for future investigations rather than as certainties requiring further research. The authors rightly emphasize that the data are cross-sectional and that causal inferences cannot be drawn; they explicitly state that “we cannot assume causal or directional associations.” However, causal language still appears intermittently, for example in phrases such as “the impact of liberal political climates on conservative individuals has significant implications” in the abstract. I would recommend revising the manuscript to replace such language with more neutral formulations (“association,” “pattern,” “relationship”) and to avoid implying that climates influence or exert effects on individuals in the absence of longitudinal or quasi-experimental evidence.

**Author response: Thank you for noting this, we have adjusted the language in the abstract accordingly.**

This is not to suggest that this is a fatal flaw that absolutely precludes publication. The article is well-argued, follows academic standards, and offers new, interesting, and partly original positions and arguments, as an article should. As mentioned, I would recommend a light revision, with particular attention to clarifying the modest incremental contribution and broadening and deepening the discussion of limitations.

**Author response: Thank you for your feedback and taking the time to review our revised manuscript. Your careful attention to detail has resulted in a significantly improved manuscript.**

To ensure your figures meet our technical requirements, please review our figure guidelines: <https://journals.plos.org/plosone/s/figures>

You may also use PLOS’s free figure tool, NAAS, to help you prepare publication quality figures: <https://journals.plos.org/plosone/s/figures#loc-tools-for-figure-preparation>.

NAAS will assess whether your figures meet our technical requirements by comparing each figure against our figure specifications.

**Author response: We have loaded our figures into the NAAS tool and have uploaded the figures the tool fixed to meet the technical requirements.**
